# Single-plate versus double-plate comparison in the surgical treatment of comminuted clavicle fractures: Is the secondary plate reliable?

**DOI:** 10.1097/MD.0000000000036711

**Published:** 2023-12-22

**Authors:** Ahmet Yurteri, Numan Mercan, Kadir Gem, Abdulkadir Bilgiç, Mehmet Kiliç, Fatih Doğar

**Affiliations:** a Department of Orthopaedic and Traumatology, Konya City Hospital, Konya, Turkey; b Department of Orthopaedic and Traumatology, Necip Fazil City Hospital, Kahramanmaras, Turkey; c Department of Orthopaedic and Traumatology, Manisa Alaşehir State Hospital, Manisa, Turkey; d Department of Orthopaedic and Traumatology, Manisa City Hospital, Manisa, Turkey; e Department of Orthopaedic and Traumatology, Faculty of Medicine, Kahramanmaras Sutcu Imam University, Kahramanmaras, Turkey.

**Keywords:** comminuted, dual, mini fragment, nonunion, shoulder

## Abstract

The objective of this study is to retrospectively assess the use of single-plate (SP) and double-plate (DP) fixation in the open reduction and internal fixation of comminuted clavicle fractures, focusing on fracture union and complications. We retrospectively evaluated comminuted diaphyseal clavicle fractures (Arbeitsgemeinschaft für Osteosynthesefragen type 15.B1-3) treated with open reduction and internal fixation and having a minimum 1-year follow-up. Two patient cohorts were identified: DP (utilizing a superiorly located clavicle-specific plate and an anteriorly located tubular plate) and SP (utilizing a superiorly located clavicle-specific plate). These groups were compared in terms of union time, peri-incisional numbness, implant irritation, return to work time, union rates, re-operation rates, Disabilities of Arm, Shoulder and Hand (DASH), and American Shoulder and Elbow Surgeons (ASES) scores. The study included 27 SP and 23 DP patients meeting the inclusion criteria. There was no significant difference between the 2 cohorts in terms of union time, peri-incisional numbness, implant irritation, return to work time, union rates, re-operation rates, DASH, and ASES scores at the end of the first year (*P* = .889, *P* = 1.00, *P* = .122, *P* = 1.00, *P* = 1.00, *P* = .493, *P* = .736, *P* = .762, *P* = .937 respectively). However, it was observed that the DP group showed a significantly earlier return to work time and better DASH scores at 3rd and 6th months, whereas the SP group exhibited significantly better ASES scores at 3rd and 6th months (*P* = .034, *P* = .016, *P* = .032, *P* = .036, *P* = .021, respectively). No significant difference was observed in terms of union and complications in acute clavicle fractures treated with SP and DP fixation. The DP group demonstrated an earlier return to work and superior early functional scores compared to the SP group. Our findings suggest that a secondary plate can be reliably used, particularly in situations where clavicle fracture fixation is insufficient or in cases of comminuted clavicle fractures.

## 1. Introduction

Clavicle fractures, which constitute 2.6% to 5% of adult fractures and 35% of shoulder girdle fractures, are among the traumas frequently encountered by orthopedic surgeons in their routine practice.^[1]^ Making connection between appendicular and axial skeleton and being close to skin are the reasons for this frequent fracture. Although majority of these fractures heal with conservative treatment, it has been reported that there are non-unions, which can reach 15% with conservative treatment.^[2]^ Although this rate may decrease to 3.7% to 4.6% with surgical treatment, non-unions or malunions can cause permanent pain, decrease in range of motion in shoulder joint and loss of workload.^[3]^

One of the reasons for nonunion in primary osteosynthesis is inadequate stabilization of the fracture and excessive micromotion after fixation.^[4]^ The use of a secondary plate in fracture fixation can enhance rigidity and provide absolute stability. Promising results have been observed with the application of double-plate (DP) in the treatment of clavicle fractures with nonunion.^[5]^ However, there is insufficient literature data on the use of DP in the surgical treatment of acute clavicle fractures. Our study aims to assess the impact of DP and single-plate (SP) utilization on clinical outcomes in acute comminuted clavicle fractures.

## 2. Material and Methods

### 2.1. Ethical statement

The study was approved by the Ethics Committee of Necmettin Erbakan University with decision number 2023/4391. The study is based on the Declaration of Helsinki and subsequent amendments. All patients signed an informed consent form prior to participation in this study.

### 2.2. Study and setting

Clavicle fractures operated in 3 distinct trauma centers between January 1, 2017 and January 1, 2022 were analyzed. Surgical fixation of the clavicle was indicated in cases where there was no contact between fracture ends (at least 100% displacement) and more than a 2 cm discrepancy on the fractured side compared to the contralateral side. The classification of clavicle fractures with a surgical indication was designated as 15.2 B1-3 fracture type according to the Arbeitsgemeinschaft für Osteosynthesefragen classification. All surgeries were conducted by 3 different orthopedic surgeons, each with a minimum of 5 years of trauma surgery training. Patients meeting the inclusion criteria were categorized into 2 groups: single-plate (SP) and double-plate (DP).

### 2.3. Survey items

**Figure 1.**
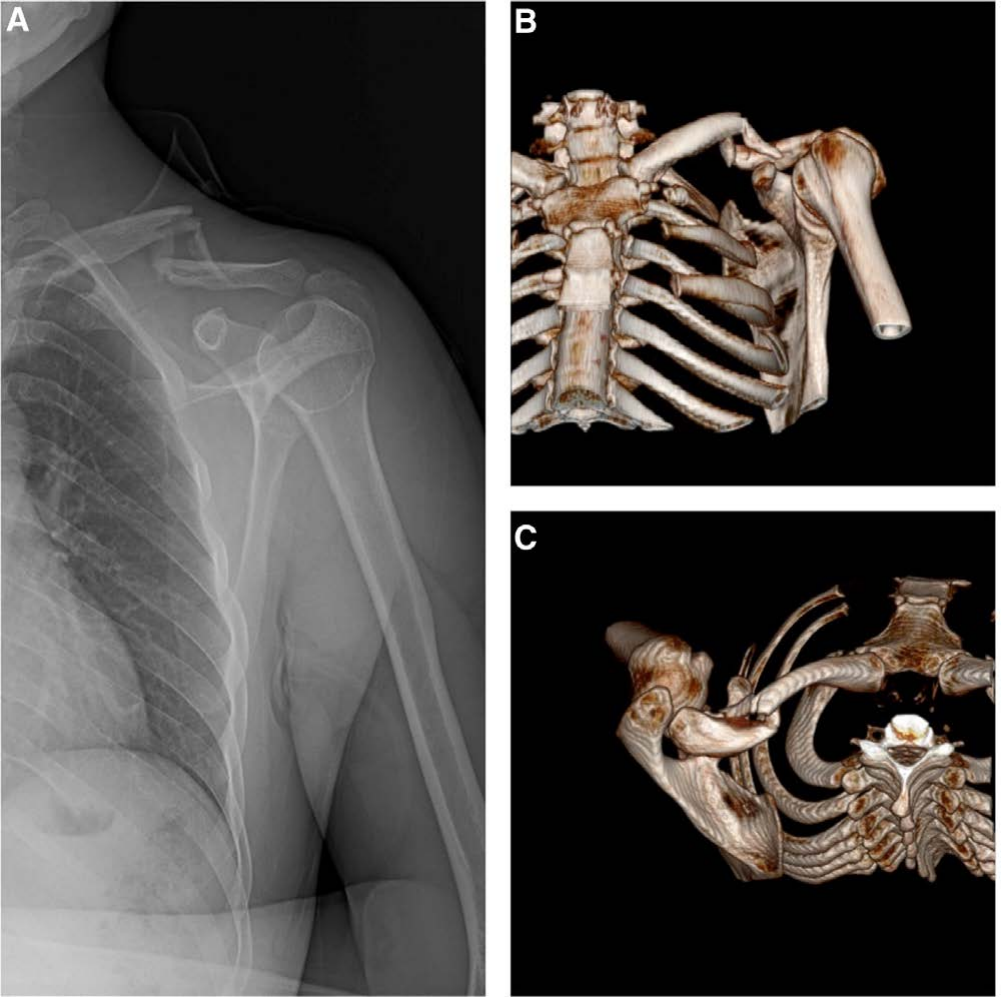
(A) Comminuted fracture of the clavicle, 30 years old, male patient, who fell from a height of about 2 meters, preoperative X-ray image. (B and C) 3-D computed tomography imaging for surgical planning.

The study examined various factors including age, gender, fractured side, smoking status, trauma severity, union rates, peri-incisional numbness, implant irritation, time to return to work, union times, re-operation rates, and functional scores in both the SP and DP groups. Functional scoring was based on the postoperative Disabilities of Arm, Shoulder, and Hand (DASH)^[6]^ and American Shoulder and Elbow Surgeons (ASES)^[7]^ scores at the end of the 1st month, 3rd month, 6th month, and 1st year. DASH scores range from 0 to 100, with 0 indicating no disability and 100 indicating the most severe disability. ASES scores also range from 0 to 100, with 0 indicating a worse shoulder condition and 100 indicating a better shoulder condition. Confirmation of union was assessed through radiographic bone bridge formation (Fig. 1) and palpation, checking for tenderness and pain at the fracture area during postoperative follow-up examinations.

### 2.4. Patient selection criteria

The inclusion criteria for the study were defined as follows: Closed fracture, absence of fractures in other areas of the shoulder girdle, no pathological findings in the upper extremity neurovascular examination, and no history of previous operations related to clavicle fractures. Exclusion criteria encompassed patients immobilized due to multiple trauma, pathological fractures, individuals with bilateral clavicle fractures, those exhibiting pathological findings in the upper extremity neurovascular examination, and patients who opted not to participate in the study.

### 2.5. Operative procedure and postoperative care

**Figure 2.**
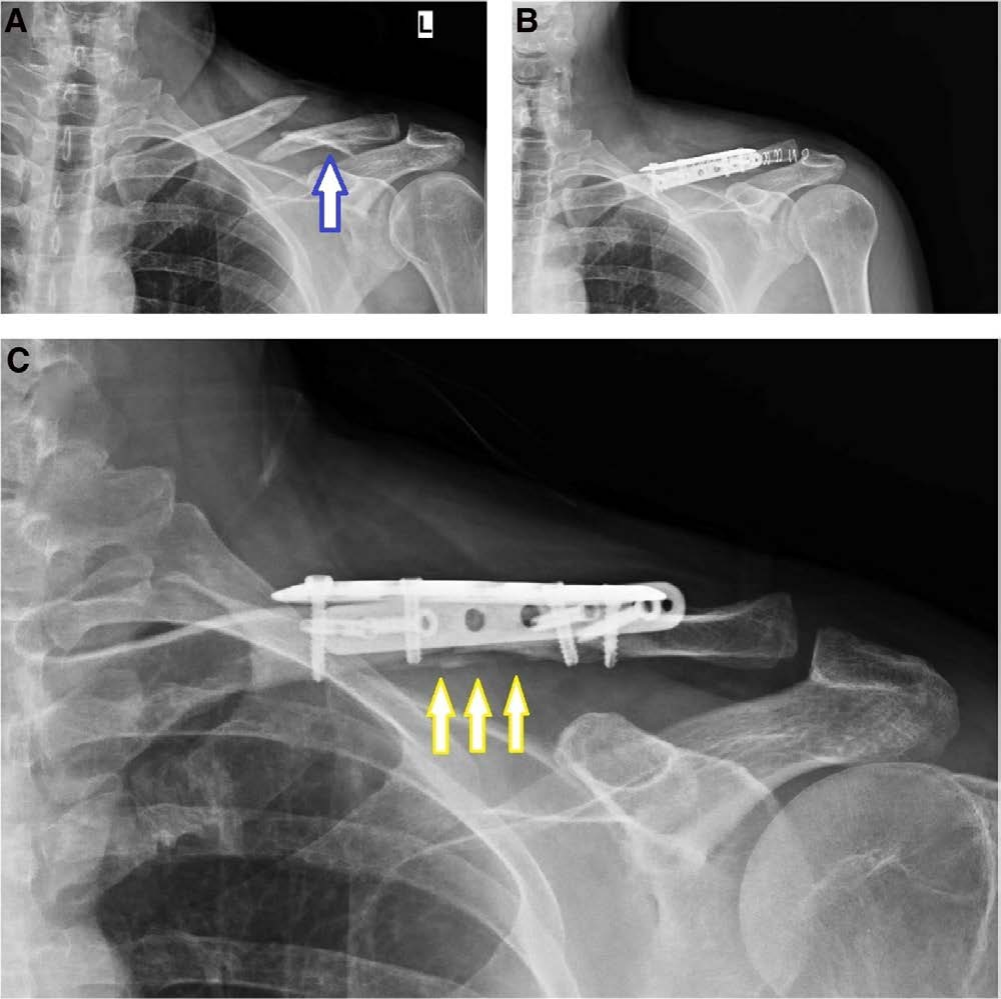
(A) Preoperative X-ray of the 45 years old, male patient, multi-fragmented clavicle fracture; blue arrow shows comminuted fracture fragment. (B) First postoperative X-ray of the patient, fracture fixation was achieved with plates placed on the superior and anterior of the clavicle. (C) Patient’s X-ray at 3rd month postoperatively; yellow arrow shows the callus bridge at the fracture line.

**Figure 3.**
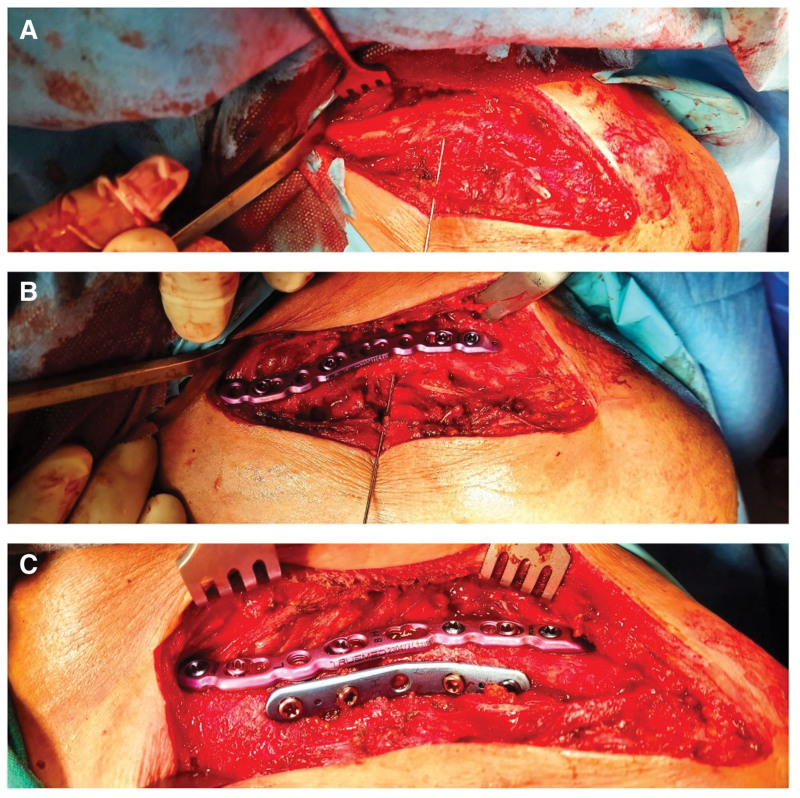
(A) Open reduction of the fracture with Kirschner wire, the periosteum on the bone is preserved and its blood supply continues (30 years old, male patient). (B) The superior locking plate was placed first. (C) The tubular plate was then placed anteriorly.

**Figure 4.**
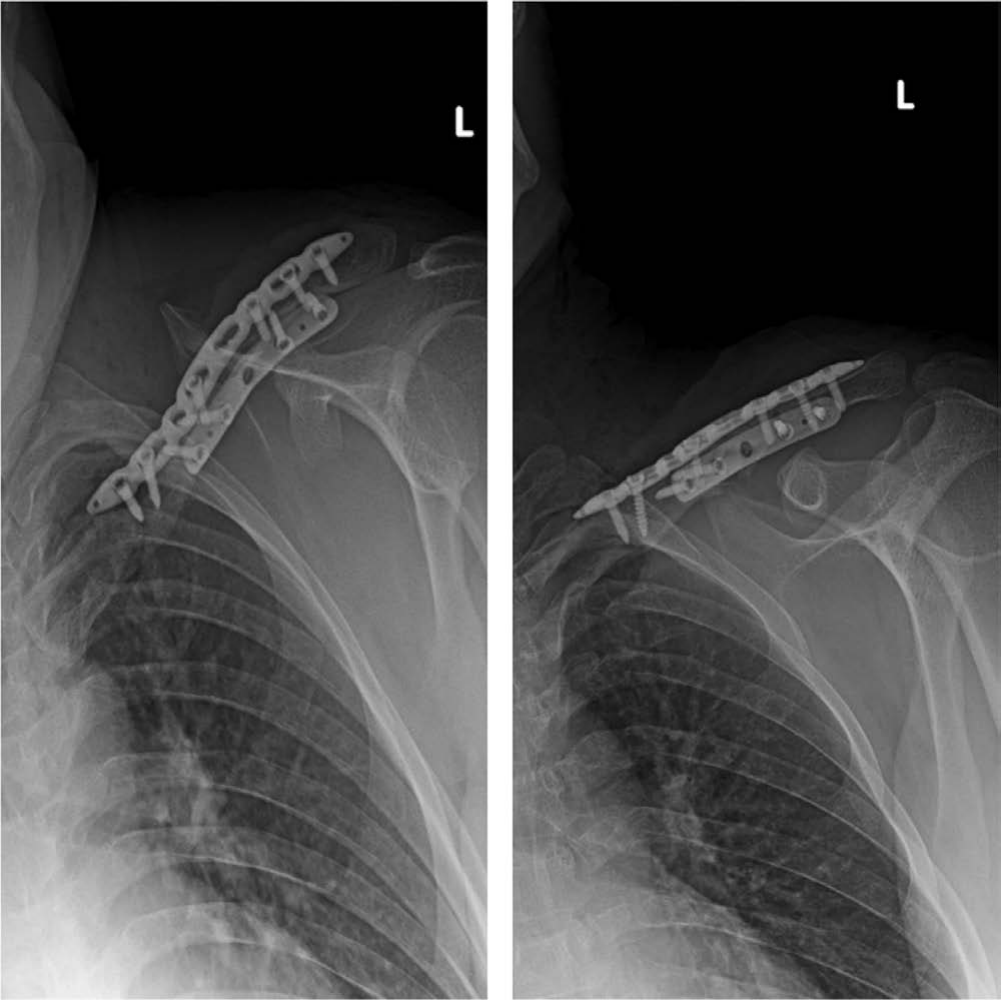
Postoperative X-rays of the patient with double-plate (30 years old, male patient).

Three-dimensional computed tomography scans were obtained for preoperative surgical planning in patients deemed necessary (Fig. 2). General anesthesia was chosen for its quality and standardization of reduction in all surgeries, which were performed with patients in the beach chair position. A prophylactic dose of 1 g EQIZOLIN (Tüm Ekip Pharmaceuticals A.Ş. Tuzla-İstanbul) antibiotic was administered to all patients at least 1 hour before the skin incision. For patients using SP (TRUEMED Medical Products Production and Marketing A.Ş. Ümraniye-İstanbul), fracture fixation was achieved by placing an anatomical clavicle plate located superiorly to the clavicle. In patients using DP (TRUEMED Medical Products Production and Marketing A.Ş. Ümraniye-İstanbul), fracture fixation involved placing a tubular plate located anteriorly to the clavicle in addition to the anatomical clavicle plate located superiorly to the clavicle (Figs. 3 and 4). Surgeons in both methods emphasized performing soft tissue exposure with minimal impact on the periosteum.

Patients were prescribed an arm sling for the first 10 days, starting from the first day postoperatively. From the 10th day to the 2nd month postoperatively, shoulder joint range of motion exercises were implemented, limited to flexion not exceeding 90° and abduction not exceeding 45°. Extremity weight-bearing and strengthening exercises were initiated at the end of the 2nd postoperative month, coinciding with clinical and radiographic improvement. Patients who demonstrated clinical and radiological improvement, along with the ability to bear weight on their extremities without pain, were given the option to return to work, depending on their profession.

### 2.6. Statistical analysis

SPSS 26.0 (Statistical Package for the Social Sciences) program was used for data analysis (Graph Pad Software, Inc., La Jolla, CA). A post hoc power analysis with an alpha of 0.05 demostrated a power of 80% for this study. Categorical variables, including gender, fracture side, smoking, trauma severity, union rate, implant prominence, peri-incisional numbness, and reoperation rate, were assessed using Fisher exact test and chi-square test. To assess the normality distribution of continuous variables (age, union times of fracture, return to work, DASH, and ASES scores at postoperative 1st, 3rd, 6th, and 12th months), the Shapiro–Wilk test was initially performed. Since the data did not fit a normal distribution, the Mann–Whitney U test was employed for comparisons between the 2 groups. The minimum, maximum, median, mean and standard deviation values of these parameters were recorded, with the level of significance set at a *P* value < .05 for all tests.

## 3. Results

The radiological and clinical scores of 50 patients who met the inclusion criteria were analyzed. No follow-up loss occurred, and clinical assessments were conducted at the postoperative 1st, 3rd, 6th months and at the end of the first year. Fracture fixation was performed using SP (3.5-mm locking plate-superior) in 27 patients and DP (3.5-mm locking plate-superior and tubular plate-anterior) in 23 patients. The mean age for SP was 42.14 ± 12.23, and for DP, it was 46.56 ± 15.87. No significant differences were found between the 2 groups in terms of age, gender, fractured side, smoking and trauma severity (*P* = .397, *P* = 1.00, *P* = .441, *P* = .297, *P* = .588, respectively; Tables 1 and 2).

**Table 1:** Categorical variables (data presented as n (%)).

	Single plate group (n = 27)	Double plate group (n = 23)	*P* value
Gender			
Male	24 (88.9)	20 (87.0)	1.00
Female	3 (11.1)	3 (13.0)	
Fracture side			
Right	10 (37)	11 (47,8)	.441
Left	17 (63)	12 (52.2)	
Trauma severity			
Low energy trauma	1 (3.7)	2 (8.7)	.588
High energy trauma	26 (96.3)	21 (91.3)	
Smoking	9 (33.3)	11 (47.8)	.297
Union rate	92.5%	100%	.493
Peri-incisional numbness	4 (14.8)	4 (17.4)	1.00
Implant irritation	4 (7.7)	5 (21.7)	.122
Reoperation rate	6 (22.2)	4 (17.4)	.736

**Table 2:** Continuous variables (data presented as mean ± SD and median).

	Single-plate group (n = 27)		Double-plate group (n = 23)		*P* value
	Mean ± SD	Median	Mean ± SD	Median	
Age	42.14 ± 12.23	42	46.56 ± 15.87	44	.397
Union time (mo)	4.92 ± 2.51	3	4.82 ± 2.16	6	.889
Return to work time (wk)	12.92 ± 6.18	12	10.69 ± 5.92	9	.034*
DASH score (postoperatively)					
1st month	68.29 ± 7.87	66.7	64.82 ± 11.47	63.3	.109
3rd month	50.35 ± 14.15	47.5	41.62 ± 7.61	41.7	.016*
6th month	31.08 ± 10.24	27.5	26.12 ± 10.7	24.2	.032*
12nd month	12 ± 5.31	10.8	10.93 ± 5.98	11.7	.762
ASES score (postoperatively)					
1st month	31.25 ± 6.4	33	30.73 ± 9.84	28	.544
3rd month	50.77 ± 9.45	53	55.08 ± 13.24	58	.036*
6th month	65.66 ± 10.14	65	71.39 ± 11.15	73	.021*
12nd month	81.07 ± 13.49	87	83.39 ± 10.80	87	.937

ASES = American Shoulder and Elbow Surgeons, DASH = Disabilities of Arm, Shoulder and Hand, SD = standard deviation

* *P* < .05.

**Figure 5.**
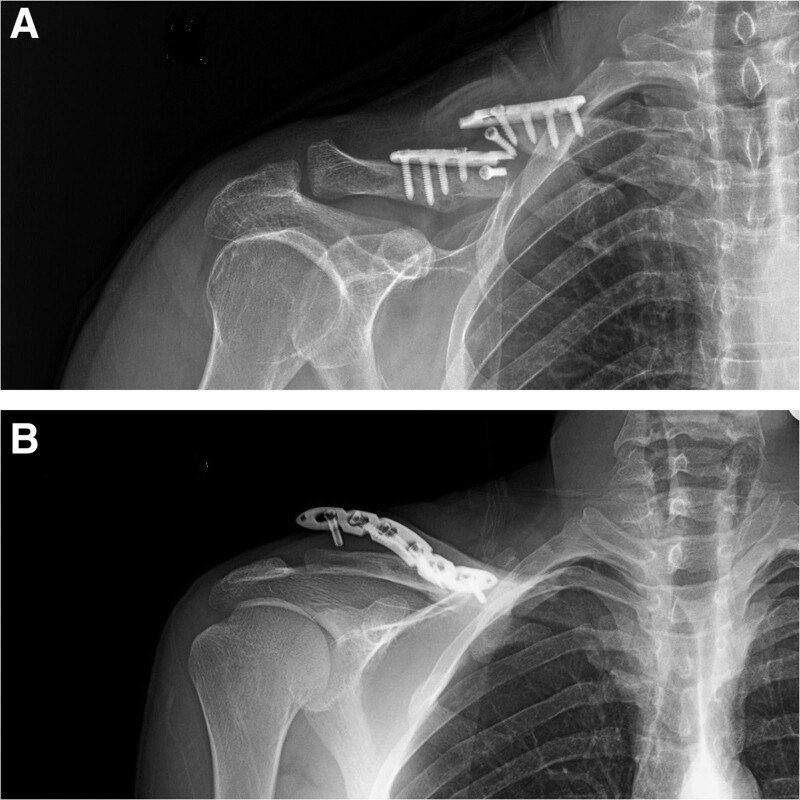
(A) X-ray of implant failure (breakage) at 3rd month postoperatively, 58 years old, male. (B) X-ray of implant failure at 2nd month postoperatively, 63 years old, male.

Of the 27 patients in the SP group, non-union and implant failure occurred in 2 (7.4%) patients (Fig. 5). Union was observed in the remaining 25 patients at the end of 1 year, with a mean time to union of 4.92 ± 2.51 months. Union was clinically and radiologically observed in 48% of patients at the 3rd month follow-up, 40.7% at the 6th month, and 7.4% at the 12th month. In the DP group, union was observed in all patients at the end of the first year, with a mean time to union of 5.18 ± 3.48 months. Clinically and radiologically, union was observed in 47.8% at the 3rd month follow-up, 47.8% at the 6th month, and 4.3% at the 12th month. No significant differences were found between the 2 groups in terms of union rate and union time (*P* = .493, *P* = .889, respectively) (Table 1).

The mean time to return to work was 12.92 ± 6.18 weeks for SP and 10.69 ± 5.92 weeks for DP. Return to work time was significantly lower in the DP group (*P* = .034). At the end of the first year, implant irritation complaints were detected in 7.7% of the SP group and 21.7% of the DP group, with no significant difference between the 2 groups (*P* = .122). Implants were removed in all SP cases with this complication, while in the DP group, implants were removed in 4 cases, and 1 patient opted against re-operation. Sensory symptoms around the incision were detected in 14.8% of SP patients and 17.4% of DP patients, with no significant difference between the 2 groups (*P* = 1.00; Tables 1 and 2).

No significant differences were found between the 2 groups in terms of DASH scores at the postoperative 1st month (*P* = .109). However, postoperative 3rd and 6th month DASH scores were significantly higher in SP patients compared to DP patients (*P* = .016, *P* = .032, respectively). No significant differences were found in DASH scores at the postoperative 12th month (*P* = .762). Similarly, there were no significant differences between the 2 groups in ASES scores at the postoperative 1st month (*P* = .544). Postoperative 3rd and 6th month ASES scores were significantly higher in DP patients compared to SP patients (*P* = .036 and *P* = .021, respectively). No significant differences were found in ASES scores at the postoperative 12th month (*P* = .937) (Table 2).

Six patients (22.2%) in the SP group underwent a secondary operation, while 4 patients (17.4%) in the DP group underwent a secondary operation. There was no significant difference in the re-operation rate between the 2 groups (*P* = .736). In the SP group, 4 patients underwent re-operation for implant removal, and 2 had re-operation due to mechanical failure. All 4 patients in the DP group were re-operated for implant removal (Table 1).

## 4. Discussion

Although the majority of clavicle fractures are treated with conservative methods, surgical intervention provides increased union rates, earlier return to work, and better shoulder joint range of motion.^[6–8]^ However, reports indicate re-operation rates ranging from 8.1 to 53% following SP open reduction and internal fixation.^[9–12]^ One of the reasons for these re-operations is implant failure. There are studies reporting that the implant failure rate is between 12% and 13.6% after internal fixation of clavicle fractures.^[13–15]^ The use of internal fixation with DP to strengthen the fixation of acute clavicle fractures and reduce implant failure rates is a popular alternative method in recent years, as it has been applied in humeral fractures, fibular fractures and fractures around the knee.^[16–19]^ Nevertheless, employing DP introduces challenges such as increased soft tissue dissection, reduced blood supply for fracture healing, heightened infection risk due to greater implant load, and concerns about nonunion.

Our study results revealed no significant differences between the SP and DP groups in terms of union rate, re-operation rate, union time, peri-incisional numbness, implant irritation complaints, and clinical functional scoring at the end of the first year. Notably, the DP group exhibited superior early clinical findings (postoperative functional scores at 3rd and 6th months) and a shorter time to return to work. However, by the end of the first year, clinical findings showed no significant disparity between the 2 groups. This suggests that patients with DP adapted more effectively to early passive-active movement and early weight-bearing exercises during postoperative rehabilitation.

Chen et al presented the radiological results of SP and DP use in a large group of patients including 188 clavicle fractures over a 15-year period.^[20]^ DP was applied to 41 patients, none of these patients underwent re-operation, and they reported that union occurred in all of the patients at the end of the 6th month. They also stated that there was no significant difference between the SP and DP groups in terms of complications, and that a secondary support plate could be used safely, similar to our study. However, in their study, besides the use of locking plates, reconstruction plates and tubular plates in different combinations for fracture fixation, these plates were placed in different combinations superior or anterior to the clavicle. We think that this may reduce the standardization of the study and affect the results. In our study, a locking plate was placed on the superior and a tubular plate was placed on the anterior of the clavicle in all patients with DP.

Another technique used in recent years is the superior and anterior fixation of the clavicle with 2 mini fragment plates (2.7 mm, 2.4 mm, or 2.0 mm) instead of fixation with a 3.5 mm SP (Locking Compression Plate, Limited Contact Dynamic Compression Plate or anatomical locking plates). It has been claimed that this method will cause less implant irritation, decrease re-operation rates, and that fixation with this method is biomechanically superior to fixation with SP.^[21]^ The low profile dual plate technique was mainly used to reduce the complaints and re-operations due to implant irritation. In our study, the results of placing tubular plates in addition to anatomical clavicle plates were evaluated. The common point of low profile DP implantation and the patients in our study group is the concerns of nonunion and infection due to the high implant load and the fact that the soft tissue dissection is slightly more for the placement of the secondary implant. Rompen et al, in their meta-analysis study on this subject, reported that the low profile DP had lower implant-related complications compared to a 3.5 mm SP, and the union and infection rates were similar.^[22]^ In our study, no significant difference was observed between the SP and DP groups in terms of union and infection. Moreover, it is promising that patients with DP have better time to return to work and better clinical outcomes in the early period. Based on this, we believe that in cases where fracture stability with a SP is not sufficient or more rigid fixation is desired, the concerns of union and infection are unfounded.

In a study by Kaipel et al examining DP of lateral clavicle fractures on 11 patients, it was reported that union was observed in all of the patients.^[23]^ However, in 2 patients, it was stated that the implants were removed due to the migration of the screws. In our study, it is seen that re-operation due to screw migration was not performed. Due to the unique anatomical structure of the distal clavicle, we believe that the application of a secondary plate to the distal clavicle from the anterior is more prone to mechanical complications than the application of a secondary plate to the clavicle shaft from the anterior.

We think that the reason for nonunion and implant failure in 2 patients in the SP group was that the fracture stabilization was not sufficient. In our study, primary ossification was aimed through rigid fixation in clavicle fractures in which both single and double-plates were used. Primary ossification is a form of fracture healing in which there is no cartilage model and requires a longer process than secondary ossification. Accordingly, we need more durable implant applications during this healing process, which requires a longer period of time. In terms of stronger and more durable implant application for comminuted clavicle fractures, we think that it may be possible to meet these conditions with the use of secondary implants, since we cannot use thicker profile implants (such as 4.5 mm plates) due to the thin skin structure and the anatomical architecture of the bone. The fact that no implant failure or bone nonunion occurred in any of the patients using DP supports that this approach is an effective method.

There are a number of articles, including published meta-analysis studies, in the existing literature regarding the use of double-plates. However, the hypothesis on which our study focuses is that a secondary plate can be used reliably for fracture stabilization in comminuted clavicle fractures. In particular, while there were 2 patients with implant failure in our study who were operated on with a single-plate, implant failure did not occur in patients operated on with double-plates. Considering the anatomy of the clavicle area, concerns may also be raised that the use of double-plates may cause skin irritation. However, in our study, there was no statistically significant difference between the 2 groups in terms of the number of patients requiring secondary surgical intervention due to skin irritation, allowing us to conclude that these concerns were unfounded. On the other hand, early pain relief due to increased fracture stabilization and shorter return to work time are among the advantages of using double-plates in the surgical treatment of comminuted clavicle fractures.

Our study has certain limitations. To begin with, as it is retrospective in nature, the pool of patients meeting the inclusion criteria is relatively small. A more extensive patient sample would unquestionably enhance the study’s robustness. Additionally, a secondary limitation is that the patients included in the study underwent surgery performed by 3 different surgeons. Although we think that it would be more appropriate for all patients to be operated by a single surgeon, we believe that this factor will not affect the outcome much, since all 3 surgeons have sufficient experience and work with the principle of minimal damage to important soft tissue supports such as the periosteum around the fracture. An additional limitation is that we assessed bone union at the fracture site using X-ray imaging instead of computed tomography imaging. This decision was driven by ethical considerations regarding the potential radiation load on the patients.

## 5. Conclusion

In our examination of acute clavicle fractures treated with both SP and DP fixation, no noteworthy disparity emerged concerning union and complications. Notably, individuals with DP demonstrated an earlier return to work and exhibited superior early clinical functional scores compared to those treated with SP. Our findings suggest that a secondary plate can be reliably used, particularly in situations where clavicle fracture fixation is insufficient or in cases of comminuted clavicle fractures.

## Acknowledgments

The authors are grateful to all participating patients.

## Author contributions

**Conceptualization:** Ahmet Yurteri, Numan Mercan, Mehmet Kiliç, Fatih Doğar.

**Data curation:** Ahmet Yurteri, Numan Mercan, Kadir Gem, Abdulkadir Bilgiç, Mehmet Kiliç, Fatih Doğar.

**Formal analysis:** Ahmet Yurteri, Numan Mercan, Fatih Doğar.

**Investigation:** Ahmet Yurteri, Numan Mercan, Kadir Gem, Abdulkadir Bilgiç, Fatih Doğar.

**Methodology:** Ahmet Yurteri, Numan Mercan, Mehmet Kiliç, Fatih Doğar.

**Project administration:** Ahmet Yurteri, Numan Mercan, Fatih Doğar.

**Resources:** Ahmet Yurteri, Numan Mercan, Kadir Gem, Abdulkadir Bilgiç, Fatih Doğar.

**Software:** Ahmet Yurteri, Numan Mercan, Mehmet Kiliç, Fatih Doğar.

**Supervision:** Ahmet Yurteri, Numan Mercan, Fatih Doğar.

**Validation:** Ahmet Yurteri, Numan Mercan, Abdulkadir Bilgiç, Mehmet Kiliç, Fatih Doğar.

**Visualization:** Ahmet Yurteri, Numan Mercan, Kadir Gem, Abdulkadir Bilgiç, Fatih Doğar.

**Writing – original draft:** Ahmet Yurteri, Numan Mercan, Fatih Doğar.

**Writing – review & editing:** Ahmet Yurteri, Numan Mercan, Kadir Gem, Abdulkadir Bilgiç, Fatih Doğar.
